# Clinical and Surgical Risk Factors for Wound Healing Disorders Following Non-Instrumented Lumbar Spine Surgery Using Penalized Regression Analysis

**DOI:** 10.3390/jcm15145467

**Published:** 2026-07-13

**Authors:** Vitalij Zeiser, David Nigl, Lukas Kohlmaier, Karl Rössler, Johannes Herta, Fabian Winter

**Affiliations:** Department of Neurosurgery, Medical University of Vienna, 1090 Vienna, Austria; vitalij.zeiser@meduniwien.ac.at (V.Z.); n12116425@students.meduniwien.ac.at (D.N.); lukas.kohlmaier@meduniwien.ac.at (L.K.); karl.roessler@meduniwien.ac.at (K.R.); johannes.herta@meduniwien.ac.at (J.H.)

**Keywords:** lumbar spine surgery, wound healing disorders, risk factor analysis, LASSO regression, Firth logistic regression

## Abstract

**Background/Objectives**: In this study, we aimed to identify clinical and laboratory factors associated with wound healing disorders (WHDs) in exclusively non-instrumented lumbar spine surgery using penalized regression analysis. **Methods**: A retrospective cohort study was conducted including 1269 adult patients undergoing non-instrumented lumbar spine surgery between 2015 and 2023. The primary outcome was postoperative WHD requiring medical or surgical intervention. Univariate logistic regression analysis was performed to identify candidate variables. Variable selection was conducted using least absolute shrinkage and selection operator (LASSO) regression, followed by Firth-corrected logistic regression. Model performance was assessed using receiver operating characteristic (ROC) curve analysis and calibration metrics. **Results**: WHD occurred in 34 patients (2.7%). Patients with WHD were significantly older and had longer operative durations (both *p* < 0.001), as well as significantly prolonged hospital stays (17.0 ± 9.9 vs. 8.0 ± 4.7 days, *p* < 0.001). In the final penalized model, age (OR 1.03, 95% CI: 1.00–1.07; *p* = 0.033) and operative duration (OR 1.01, 95% CI: 1.01–1.02; *p* < 0.001) were independently associated with WHD, while arterial hypertension showed a borderline association (OR 2.28, 95% CI: 0.96–6.04; *p* = 0.063). The model demonstrated moderate discriminative performance with an area under the curve (AUC) of 0.795 (95% CI: 0.72–0.87). **Conclusions**: Wound healing disorders following non-instrumented lumbar spine surgery are multifactorial. While several patient- and surgery-related variables were associated with wound healing disorders individually, only advanced age and longer operative duration remained independently associated with WHD after adjustment for multiple variables.

## 1. Introduction

Low back pain affects up to 80% of the population and represents a major cause of disability worldwide [1,2]. Among the most common surgically treated etiologies are lumbar disc herniation and lumbar spinal canal stenosis [1,2]. Microsurgical disc excision and lumbar decompression procedures, including laminectomy and minimally invasive decompression techniques, are widely used for the treatment of these conditions [1,2]. Although these procedures are generally considered safe, they are associated with perioperative complications such as recurrent disc herniation, postoperative infection, and wound healing disorders (WHDs) [3].

WHDs remain clinically relevant because they are associated with increased morbidity, prolonged hospitalization, reoperation, and healthcare burden in spine surgery cohorts [4,5]. Risk factors for wound complications and surgical site infections after spine surgery have been widely investigated and include patient-related and systemic factors such as advanced age, obesity, diabetes mellitus, smoking, hypertension, steroid use, malnutrition, hypoalbuminemia, anemia, and renal dysfunction, as well as procedure-related factors such as longer operative duration, greater surgical complexity, multilevel surgery, blood loss, transfusion, revision surgery, and instrumentation [6,7,8,9,10,11,12,13,14,15,16]. In addition, measures of subcutaneous fat thickness have been shown to correlate with infection risk in lumbar spine surgery [17,18,19].

However, much of the available evidence is derived from heterogeneous spine surgery cohorts, frequently including instrumented fusion procedures, revision surgery, or mixed spinal regions. This limits direct applicability to non-instrumented lumbar decompression and discectomy, where implant-related factors are absent and surgical exposure is typically smaller. Although preoperative anemia and other laboratory abnormalities have been associated with adverse postoperative outcomes in general surgical and spine surgery populations, their specific contribution to WHD after exclusively non-instrumented lumbar spine surgery remains insufficiently defined [6,11,12,20]. Therefore, procedure-specific analyses are needed to clarify which clinical, surgical, and laboratory factors are associated with WHD in this common surgical population.

The aim of this study was to identify clinical, surgical, and laboratory factors associated with WHD in a large cohort of patients undergoing non-instrumented lumbar spine surgery. Given the low event rate of WHD, we additionally used penalized regression techniques to reduce the risk of model overfitting.

## 2. Materials and Methods

The study was conducted in accordance with the Declaration of Helsinki and its subsequent amendments and was approved by the local ethics committee of the Medical University of Vienna (ethics approval number 1298/2025).

This retrospective cohort study included all consecutive adult patients who underwent non-instrumented lumbar spine surgery at the Department of Neurosurgery, Medical University of Vienna, between January 2015 and December 2023. In total, 1269 patients who underwent surgery were included.

**Figure 1:**
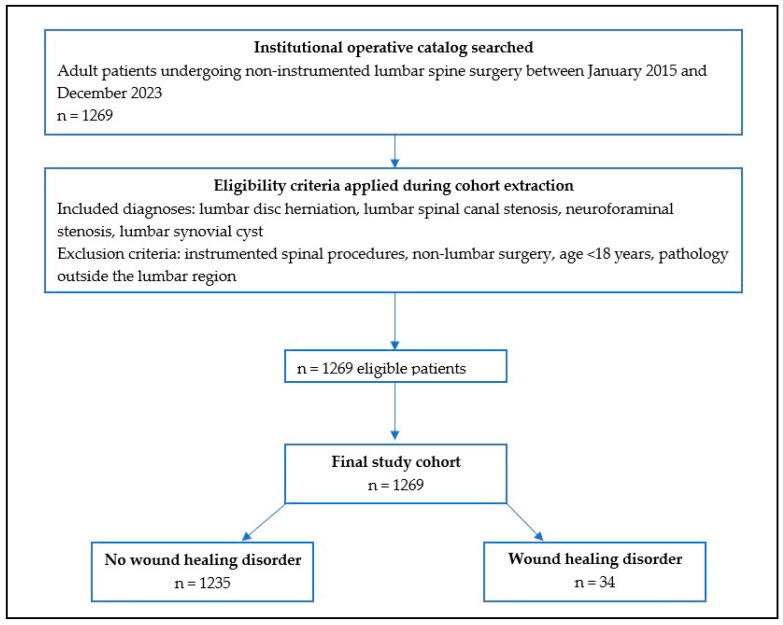
Patient selection flow diagram. Patients were identified from the institutional operative catalog using predefined inclusion and exclusion criteria. Because cohort extraction was performed directly according to these criteria, all identified cases fulfilled the eligibility criteria and no additional exclusions were made after cohort assembly. WHD: wound healing disorder.

Inclusion criteria comprised patients aged 18 years or older undergoing surgery for (1) lumbar spinal canal stenosis, (2) neuroforaminal stenosis, (3) lumbar disc herniation, or (4) lumbar synovial cyst. Exclusion criteria included spinal pathology outside the lumbar region, instrumented spinal procedures, non-lumbar spine surgery, and age younger than 18 years. Figure 1 demonstrates the patient selection flow diagram.

Demographic, clinical, surgical, and laboratory data were extracted from electronic medical records. Collected variables included age at surgery, sex, comorbidities, operative duration, and number of operated segments.

Standard preoperative laboratory parameters included blood count, coagulation parameters, clinical chemistry, C-reactive protein (CRP) and thyroid-stimulating hormone (TSH) as well as nutritional laboratory values like protein, albumin, triglyceride and total cholesterol. All laboratory values were obtained as part of routine preoperative assessment.

**Figure 2:**
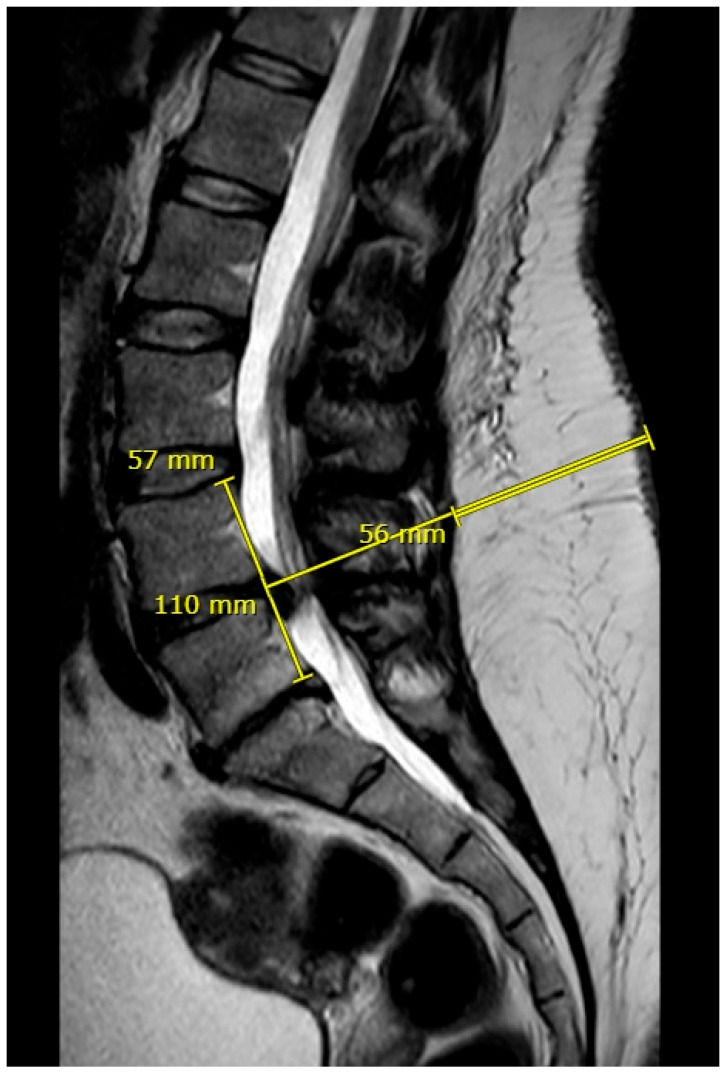
Example of LOA ratio measurement. The LOA ratio was calculated as: (short lumbosacral thickness/long lumbosacral thickness) × number of operated segments × 10. Short lumbosacral thickness was defined as the distance from the thoracolumbar fascia to the dermis in the midsagittal plane. Long lumbosacral thickness was defined as the length of a perpendicular line extending from the dorsal vertebral endplate (base of the operated segment) to the dermis. Distances are shown in millimeters (mm).

To assess local anatomical and surgical factors, a study-specific parameter termed the lumbosacral operated area (LOA) ratio was introduced in addition to operative duration and number of operated segments. Measurements were performed using standard radiological tools on sagittal magnetic resonance images (Figure 2). The LOA ratio was calculated as:

(short lumbosacral thickness/long lumbosacral thickness) × number of operated segments × 10.

Short lumbosacral thickness was defined as the distance from the thoracolumbar fascia to the dermis in the midsagittal imaging. Long lumbosacral thickness was defined as the orthogonal distance, which was measured from the dorsal vertebral end plate to the base end plate of the operated segment, to the dermis.

The rationale for the LOA ratio was to provide a simple surrogate measure of the local wound burden created by surgery. Patients with greater subcutaneous tissue thickness and/or multilevel surgery are likely to have a larger wound surface or wound volume, which may increase tissue trauma, dead space, impaired perfusion, and the risk of delayed wound healing. The LOA ratio was therefore designed as an easily obtainable imaging-based parameter to approximate the wound area affected by the surgical approach and to evaluate whether local anatomical and procedural factors influence postoperative wound healing.

Lumbar disc herniations were routinely operated using an open microsurgical approach with hemilaminotomy. Lumbar spinal stenosis was primarily treated using a unilateral microsurgical approach with bilateral trans-spinal decompression. Laminectomy was performed in selected cases but has largely been replaced by microsurgical bilateral decompression at our institution. In patients with complex multilevel pathology (e.g., combined disc herniation and lumbar spinal stenosis due to hypertrophic facet joints and ligamentum flavum) partial (hemi)laminectomy with trans-spinal bilateral decompression via unilateral approach was performed.

The primary outcome was the occurrence of postoperative WHD, which was assessed during the index hospitalization and a postoperative follow-up of 90 days documented in the institutional medical record. WHD was defined as a composite endpoint because the study aimed to capture clinically relevant wound-related complications requiring medical (e.g., antibiotics, Steri-Strips, wound dressing) or surgical intervention. The individual components of the composite endpoint included superficial or deep wound dehiscence, surgical site infection, wound abscess, postoperative hematoma requiring intervention, CSF fistula requiring treatment, and secondary wound closure.

Statistical analysis was performed using IBM SPSS Statistics (version 29.0) and R (version 4.2.1; R Foundation for Statistical Computing). Continuous variables are presented as mean ± standard deviation, categorical variables as counts and percentages, and laboratory values as median, interquartile range (IQR), and minimum and maximum values. Group comparisons between patients with and without WHD were performed using appropriate univariate statistical tests. Univariate logistic regression analysis was conducted to identify potential predictors of WHD. Variable selection was performed using least absolute shrinkage and selection operator (LASSO) regression. Missing data were handled according to the type of analysis. For univariate analyses, available-case analysis was performed, and each variable was analyzed using all patients with available data for that specific variable. For the penalized regression model, complete-case analysis was used. No multiple imputation was performed. Because preoperative laboratory parameters were incomplete and the number of WHD events among patients with available laboratory values was low, laboratory variables were analyzed exploratively in univariate analyses but were not included in the primary LASSO candidate set. Ten-fold cross-validation was used to determine the optimal penalty parameter (λ) in the LASSO model. The final model was subsequently fitted using Firth-corrected logistic regression to reduce small-sample bias due to the low number of outcome events. The availability of individual laboratory parameters is reported in Supplementary Table S1. Model discrimination was assessed using receiver operating characteristic (ROC) curve analysis. Model calibration was assessed using a calibration plot, calibration intercept, calibration slope, and Brier score. Calibration metrics were calculated based on predicted probabilities derived from the final Firth-corrected logistic regression model. Because model performance was assessed in the same cohort in which the model was developed, these metrics represent apparent model performance. A two-sided significance level (α) of 0.05 was applied, and *p*-values < 0.05 were considered statistically significant.

## 3. Results

A total of 1269 patients undergoing non-instrumented lumbar spine surgery were included in the analysis. Baseline demographic, clinical, and surgical characteristics are summarized in Table 1.

**Figure 3:**
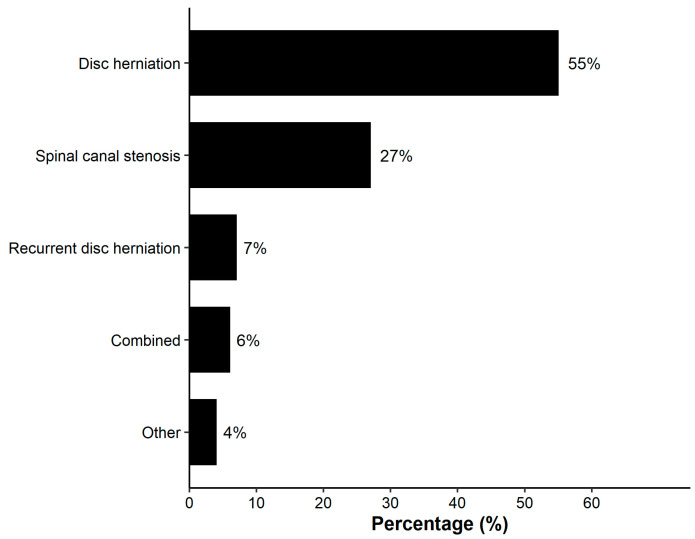
Distribution of preoperative diagnoses. “Other” includes neuroforaminal stenosis (1.1%), synovial cyst (1.9%), and recurrent spinal canal stenosis (1.0%).

**Figure 4:**
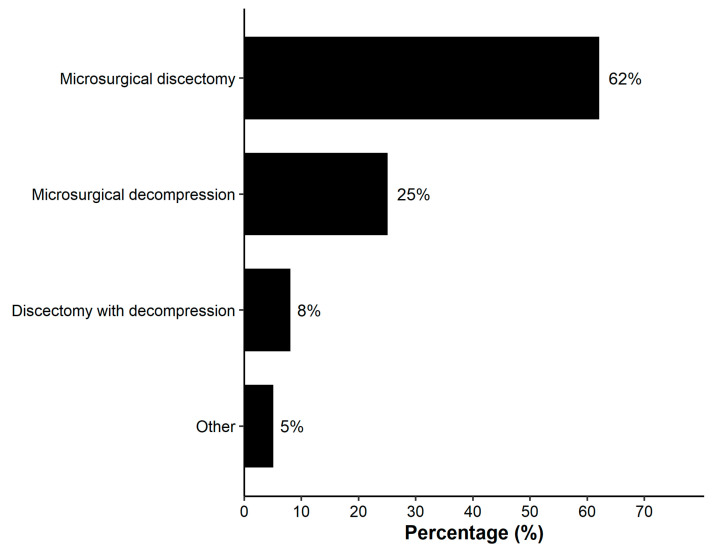
Distribution of surgical procedures. “Other” includes laminectomy (2.7%), cyst resection (1.8%), hemilaminectomy with decompression (1.6%), and laminectomy with decompression (2.0%).

Figure 3 and Figure 4 illustrate the distribution of the preoperative diagnosis and the surgical procedures. The most common diagnoses were disc herniation (55%), followed by lumbar spinal stenosis (27%), and recurrent disc herniation (7%). The most frequently performed procedure was microsurgical discectomy (62%), followed by microsurgical decompression (25%).

Postoperative WHD occurred in 34 patients (2.7%). Of these, 13/34 (38.2%) were superficial WHD limited to the dermis and subcutaneous tissue, whereas 21/34 (61.8%) were classified as deep WHD. Surgical site infection was present in 8 cases, including 5 cases of deep wound abscess.

Conservative management was performed in 10/34 (29.4%) cases, while 24/34 (70.6%) patients required surgical intervention. Surgical procedures included wound revision (3/24, 12.5%), hematoma evacuation (10/24, 41.7%), abscess drainage (3/24, 12.5%), repair of a postoperative cerebrospinal fluid (CSF) fistula (1/24, 4.2%), and secondary wound closure (5/24, 20.8%). In 2/24 (8.3%) cases, multiple surgical procedures were required.

Patients with WHD demonstrated significant differences in selected clinical and laboratory parameters compared to patients without complications. Univariate logistic regression analysis identified several variables associated with postoperative WHD (Table 2 and Table 3).

Patients with WHD were significantly older (*p* < 0.001) and had a longer duration of surgery (*p* < 0.001). Additional significant clinical variables included the number of operated segments (*p* = 0.009), preoperative active anticoagulation (defined as intake of anticoagulant medication within 48 h prior to surgery or antiplatelet medication within 5 days prior to surgery; *p* = 0.011), arterial hypertension (*p* < 0.001), endocrinological disease (including diabetes, hypothyroidism or hyperthyroidism; *p* = 0.044), type 2 diabetes (*p* = 0.007), BMI (in kg/m^2^; *p* = 0.034), and the study-specific parameter LOA ratio (*p* = 0.012). Patients with WHD had a significantly longer length of stay compared to patients without WHD (mean in days 17.0 ± 9.9 vs. 8.0 ± 4.7; *p* < 0.001).

In univariate logistic regression analysis of preoperative laboratory parameters (Table 3) erythrocyte count (*p* < 0.001), hemoglobin (*p* < 0.001), hematocrit (*p* < 0.001), platelet count (*p* = 0.046), INR (*p* = 0.025), potassium (*p* < 0.001), creatinine (*p* = 0.029), blood urea nitrogen (BUN; *p* < 0.001), albumin (*p* = 0.016), alkaline phosphatase (*p* = 0.018), aspartate aminotransferase (AST; *p* = 0.022), gamma-glutamyl transferase (GGT; *p* = 0.015), lactate dehydrogenase (LDH; *p* < 0.001), glucose (*p* = 0.005), HbA1c (IFCC; *p* < 0.001), triglycerides (*p* = 0.014), and total cholesterol (*p* = 0.030) were associated with WHD.

Given the low number of WHD events, conventional multivariable logistic regression was not performed because of the risk of model overfitting. Instead, LASSO regression followed by Firth-corrected logistic regression was applied (Table 4). Candidate predictors for the primary penalized model were selected based on clinical relevance, availability, and univariate associations, while limiting the number of predictors due to the low event rate. Candidate variables entered into LASSO regression were age, operative duration, arterial hypertension, LOA ratio, number of operated segments, and diabetes mellitus.

LASSO selected age, operative duration, and arterial hypertension. In the subsequent Firth bias-reduced logistic regression, age (*p* = 0.033) and operative duration (*p* < 0.001) remained independently associated with WHD, whereas arterial hypertension showed a borderline association (*p* = 0.063). The LOA ratio, number of operated segments and diabetes mellitus were not retained in the penalized model.

**Figure 5:**
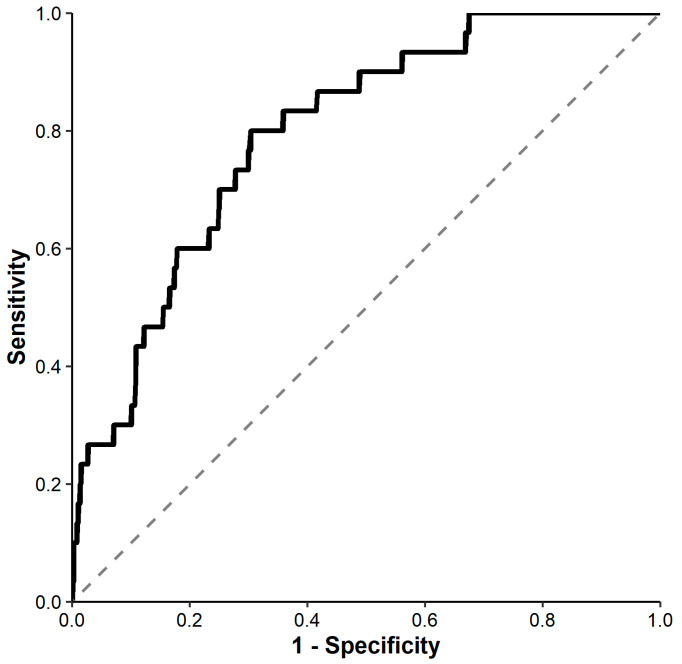
Receiver operating characteristic curve of the final Firth-corrected logistic regression model predicting postoperative wound healing disorders. AUC = 0.795 (95% CI: 0.72–0.87).

The discriminative ability of the final model was assessed using ROC curve analysis (Figure 5). The model demonstrated moderate discriminative performance, with an area under the curve (AUC) of 0.795 (95% CI: 0.72–0.87).

Calibration analysis demonstrated good apparent calibration of the final model, with a calibration intercept of 0.001, a calibration slope of 1.020, and a Brier score of 0.026. The calibration plot (Supplementary Figure S1) showed acceptable agreement between predicted and observed WHD risk, although these estimates should be interpreted cautiously given the low number of WHD events.

## 4. Discussion

In this retrospective analysis of patients undergoing non-instrumented lumbar spine surgery, advanced age and operative duration remained independently associated with postoperative WHD in the final penalized model. Arterial hypertension showed a borderline association, whereas the LOA ratio, number of operated segments and diabetes mellitus were not retained after penalized variable selection. These findings emphasize the multifactorial nature of WHD and suggest that patient vulnerability and surgical complexity may be more relevant than any single isolated risk factor.

WHDs are associated with substantial clinical and economic burden, including prolonged hospitalization, increased reoperation rates, and higher healthcare costs [4,5,8,20,21]. In spine surgery, these complications may lead to repeated surgical interventions, prolonged antibiotic therapy, and, in severe cases, systemic complications such as sepsis and increased mortality [3,4,5,8,20,21]. Previous studies have demonstrated that wound complications can double the length of hospital stay and significantly increase the likelihood of reoperation, underscoring their clinical relevance [8,20,22]. These findings are consistent with our results, which showed an approximately twofold longer length of stay in patients with WHD. The mean length of stay in patients without WHD was 8.0 days, which may appear longer than that reported in some other healthcare settings. This likely reflects institution- and healthcare-system-specific factors, including preoperative inpatient admission while awaiting surgery in selected cases, postoperative monitoring practices, rehabilitation planning, and delayed discharge due to limited social support or home-care availability. Therefore, length-of-stay data should be interpreted in the context of local healthcare structures and may not be directly generalizable to institutions with shorter routine postoperative admissions.

Operative duration showed the strongest association with WHD in the final model. This finding is consistent with previous studies showing that prolonged surgical time is associated with an increased risk of surgical site infection and wound complications after spine surgery [3,4,5,8,20,23,24].

However, operative duration should not be interpreted as an isolated causal factor, but rather as a surrogate for procedural complexity, extent of tissue dissection, blood loss, and cumulative surgical burden [3,4]. Accordingly, the association between operative duration and WHD likely reflects both the technical demands of the procedure and the degree of local tissue stress. From a clinical perspective, operative duration may therefore serve as a measurable intraoperative marker of surgical complexity and should be considered during perioperative risk assessment and procedural planning.

Advanced age remained independently associated with WHD in the final penalized model. Age-related impairment in wound healing has been well documented and is attributed to reduced vascularization, diminished immune response, and increased comorbidity burden. These findings are consistent with prior reports in spine surgery cohorts [15,23,24,25].

Recent spine surgery literature suggests that frailty is a strong predictor of adverse postoperative outcomes and may also serve as a summary indicator of SSI risk. Accordingly, the observed association between age and WHD in the present study may reflect broader frailty-related vulnerability rather than chronological age alone. This interpretation is supported by recent evidence suggesting that age may lose significance after adjustment for comorbidities, highlighting the potential importance of biological age, frailty, and systemic vulnerability in postoperative infection risk [26,27,28].

Arterial hypertension showed a borderline association with WHD in the final model. Hypertension may contribute to impaired wound healing through microvascular dysfunction, endothelial damage, and reduced tissue perfusion. While hypertension is commonly reported as a comorbidity in large surgical cohorts, its independent impact on wound complications has been inconsistently demonstrated in the literature, suggesting that its independent contribution remains uncertain and may depend on interactions with other systemic risk factors, particularly in older patients with multimorbidity [8,20,23].

Several preoperative laboratory parameters were associated with WHD in univariate analysis, particularly markers of hematologic reserve, nutritional status, renal function, and metabolic dysregulation. Lower hemoglobin, hematocrit, and erythrocyte count may reflect impaired physiological reserve or reduced tissue oxygen delivery, while lower albumin may indicate impaired nutritional status or systemic inflammation. Higher creatinine and BUN may reflect renal dysfunction or systemic vulnerability, and higher glucose and HbA1c support the role of impaired glycemic control in postoperative wound complications. Hyperglycemia and insulin resistance may impair tissue repair by promoting oxidative stress, inflammatory activation, immune dysfunction, and altered glucose metabolism, all of which are relevant components of the wound healing cascade [29]. In addition, lipid dysregulation may contribute to impaired microvascular perfusion and systemic metabolic imbalance. In this context, a recent meta-analysis of chitosan interventions demonstrated significant effects on triglycerides, total cholesterol, LDL cholesterol, body weight, and BMI, supporting the biological relevance of lipid regulation in metabolic homeostasis [30]. Recent reviews on bioactive compounds, gut microbiota, and glucose regulation further support the concept that systemic metabolic homeostasis, insulin-related pathways, and chronic low-grade inflammation are closely linked to diabetes-related tissue repair capacity [31]. These findings are consistent with broader surgical and spine literature linking anemia, malnutrition, hypoalbuminemia, diabetes, and impaired glycemic control to adverse postoperative outcomes and surgical site infection [4,5,11,12,32,33,34]. However, because laboratory data were incomplete and the number of WHD events among patients with available laboratory values was low, these findings should be considered exploratory and hypothesis-generating rather than independent predictors.

Importantly, several variables traditionally considered relevant for wound complications, including BMI, diabetes mellitus, number of operated segments, albumin, and glycemic parameters, were associated with WHD in univariate analysis but were not retained in the final penalized model or were not included in the primary LASSO candidate set because of incomplete availability. This does not imply that these factors are clinically irrelevant; rather, their effects may overlap with broader markers of patient vulnerability and surgical complexity, particularly age and operative duration. The low number of WHD events further limited the number of variables that could be reliably evaluated in the final model. These findings support cautious interpretation and reinforce the rationale for using penalized regression to reduce overfitting in a low-event-rate dataset.

The LOA ratio represents an exploratory surrogate parameter designed to approximate the local wound burden created by surgery. The underlying rationale was that a larger surgically created wound area may increase local wound burden through greater tissue trauma, increased dead space, and impaired perfusion, thereby potentially increasing the risk of delayed wound healing. Therefore, the LOA ratio was designed as an easily obtainable imaging-based parameter to estimate the affected wound area. The LOA ratio was associated with WHD in univariate analysis but was not retained after LASSO-based variable selection. This suggests that its effect may be mediated by other variables, such as operative duration and overall surgical complexity. Although measures of subcutaneous tissue thickness have previously been associated with infection risk in spine surgery, the role of combined anatomical and procedural indices remains insufficiently studied [14,17,18]. Therefore, the LOA ratio should not yet be interpreted as an independent predictor, but rather as an exploratory anatomical-procedural marker that requires further external validation, including assessment of measurement reliability, interobserver agreement, and clinical utility.

The final model demonstrated moderate discriminative performance (AUC 0.795), with good apparent calibration as indicated by a calibration intercept close to 0 and a calibration slope close to 1. However, these findings represent apparent performance within the development cohort and should not be interpreted as external validation.

In clinical practice, the results of the final model may support perioperative risk awareness rather than guide treatment decisions in isolation. Older and potentially frailer patients, as well as patients expected to undergo longer procedures, may benefit from careful procedural planning, avoidance of unnecessarily prolonged operative time, heightened postoperative wound surveillance, and structured follow-up after discharge.

This study has several limitations. The single-center retrospective design introduces potential bias and limits causal inference and generalizability. The relatively low number of events may affect statistical power and model stability, despite the use of penalized regression techniques. The composite WHD endpoint included clinically heterogeneous events, such as infection, hematoma, CSF fistula, and wound dehiscence. Although all events were wound-related and required medical or surgical management, their underlying mechanisms may differ. Additionally, some of the preoperative variables, in particular the laboratory values prior to surgery, were incomplete, and could therefore not be included in the LASSO model due to the low number of events. Furthermore, the LOA ratio was introduced as an exploratory parameter, and measurement reliability, interobserver agreement, and clinical utility were not assessed in the present study. Because missing data were handled using available-case analyses for univariate testing and complete-case analysis for the penalized model, selection bias cannot be excluded, particularly for laboratory parameters with incomplete availability. Residual confounding cannot be excluded because granular perioperative variables such as detailed nutritional assessment, intraoperative blood loss, perioperative glycemic control, antibiotic prophylaxis protocols, wound dressing protocols, and postoperative mobilization or home-care arrangements were not available in sufficient detail.

The identification of operative duration as a potentially modifiable risk factor highlights the importance of surgical efficiency and careful procedural planning. Although age is non-modifiable, it may serve as a marker of increased physiological vulnerability. While the observed odds ratios for age and operative duration were modest on a per-unit basis, these effects may become clinically relevant when accumulated across larger differences in patient age or operative duration. Furthermore, both variables likely reflect broader physiological vulnerability and surgical complexity rather than isolated causal mechanisms. Collectively, these findings support a comprehensive, patient-centered approach to perioperative risk assessment that integrates both surgical and patient-related factors.

## 5. Conclusions

WHDs following non-instrumented lumbar spine surgery are influenced by a combination of surgical and patient-related factors. While several clinical and laboratory variables were associated with WHDs in univariate analysis, only age and operative duration remained independently associated with WHDs in the final penalized model. These findings may support perioperative risk awareness and structured postoperative wound surveillance, but require external validation before clinical implementation, because the model performance was evaluated using the same cohort.

## Figures and Tables

**Table 1:** Baseline characteristics of the study cohort.

Variable	Overall Cohort (n = 1269)
Male sex	683 (53.8%)
Age, years	56.3 ± 16.2
BMI, kg/m^2^	28.0 ± 5.5
Elective operation	1070 (84.3%)
Multisegmental	143 (11.3%)
Operative duration, min	114 ± 46.0
Length of stay, days	8.3 ± 5.1
Preop anticoagulation	26 (2.0%)
Arterial hypertension	547 (43.1%)
Endocrinological disease	361 (28.4%)
Diabetes	220 (17.3%)
COPD	66 (5.2%)
Smoking	356 (28.1%)
Immunosuppressive drugs	49 (3.9%)
Wound healing disorder	34 (2.7%)

COPD: chronic obstructive pulmonary disease; Multisegmental: two or more lumbar segments were operated; Preop anticoagulation: includes antiplatelet drugs.

**Table 2:** Univariate logistic regression analysis of clinical risk factors for wound healing disorder.

Variable	No WHD	WHD	OR	95% CI	*p*-Value
Male sex	669/1235 (54.2%)	14/34 (41.2%)	1.69	0.85–3.37	0.138
Age, years	55.9 ± 16.2	67.0 ± 11.3	1.05	1.02–1.08	<0.001
Elective operation	1039/1235 (84.1%)	31/34 (91.2%)	1.95	0.59–6.44	0.274
Operative duration, min	112.8 ± 44.2	154.8 ± 71.5	1.02	1.01–1.02	<0.001
Number of segments	1.1 ± 0.3	1.2 ± 0.4	2.79	1.30–6.00	0.009
Use of hydrogen peroxide	368/1235 (29.8%)	14/34 (41.2%)	1.65	0.82–3.30	0.158
Preop anticoagulation	23/1233 (1.9%)	3/34 (8.8%)	5.09	1.45–17.86	0.011
Arterial hypertension	521/1234 (42.2%)	26/34 (76.5%)	4.49	2.00–9.90	<0.001
Endocrinological disease	346/1234 (28.0%)	15/34 (44.1%)	2.03	1.02–4.03	0.044
Diabetes	208/1234 (16.9%)	12/34 (35.3%)	2.69	1.31–5.52	0.007
COPD	63/1232 (5.1%)	3/34 (8.8%)	1.18	0.62–2.23	0.619
Smoking	351/1229 (28.6%)	5/34 (14.7%)	0.43	0.17–1.12	0.085
Immunosuppressive drugs	47/1232 (3.8%)	2/34 (5.9%)	1.58	0.37–6.77	0.541
BMI, kg/m^2^	27.9 ± 5.5	30.0 ± 5.7	1.06	1.01–1.13	0.034
Postop drain *	1022/1233 (82.9%)	25/34 (73.5%)	0.56	0.26–1.21	0.140
Postop antibiotics *	532/1229 (43.3%)	20/33 (60.6%)	1.99	0.99–4.01	0.055
LOA ratio	3.7 ± 1.8	4.6 ± 2.3	1.18	1.04–1.35	0.012

LOA ratio: lumbosacral operated area ratio: LOA ratio: (short lumbosacral thickness/long lumbosacral thickness) × number of operated segments × 10; WHD: wound healing disorder; * exploratory analysis.

**Table 3:** Univariate logistic regression analysis of preoperative laboratory parameters.

Variable	No WHD *	WHD *	OR	95% CI	*p*-Value
Erythrocyte count (T/L)	4.7 (0.7; 3.1–7.2)	4.5 (1.0; 3.0–5.3)	0.30	0.14–0.60	<0.001
Hemoglobin (g/dL)	14.1 (2.0; 8.5–20.8)	12.8 (2.9; 8.8–16.2)	0.63	0.50–0.79	<0.001
Hematocrit (%)	41.4 (5.2; 26.8–60.1)	38.1 (7.3; 26.3–46.7)	0.84	0.77–0.92	<0.001
MCV (fL)	88.0 (6.1; 56.8–105.6)	86.9 (6.0; 80.6–100.3)	0.99	0.93–1.06	0.811
MCH (pg)	30.0 (2.2; 17.9–36.4)	29.6 (2.8; 26.9–33.0)	0.91	0.75–1.10	0.315
MCHC (g/dL)	34.1 (1.5; 30.4–37.3)	33.6 (1.6; 31.5–36.9)	0.94	0.78–1.13	0.482
Platelet count (G/L)	256.0 (85.0; 77.0–634)	219.0 (107.5; 134.0–355.0)	0.99	0.99–1.00	0.046
Leucocyte count (G/L)	7.9 (3.1; 2.7–22.4)	7.2 (3.0; 3.9–12.8)	0.84	0.70–1.02	0.075
Prothrombin time (%)	97.0 (24.3; 35.2–125.0)	95.0 (31.0; 33.0–122.0)	0.98	0.96–1.01	0.130
INR	1.0 (0.2; 0.9–1.4)	1.0 (0.2; 0.9–1.5)	59.3	1.65–2129	0.025
aPTT (sec)	34.3 (4.6; 25.9–78.7)	33.9 (5.4; 28.5–43.5)	1.03	0.95–1.12	0.505
Fibrinogen (mg/dL)	330.0 (106.0; 139.0–686.0)	350.0 (107.0; 221.0–491.0)	1.00	0.99–1.01	0.136
Sodium (mmol/L)	140.0 (3.0; 124.0–147.0)	141.0 (3.0; 130.0–144.0)	0.95	0.82–1.09	0.436
Potassium (mmol/L)	4.0 (0.4; 2.8–5.6)	4.2 (0.6; 3.7–5.6)	6.49	2.33–18.1	<0.001
Creatinine (mg/dL)	0.9 (0.3; 0.4–4.6)	0.9 (0.5; 0.5–2.6)	2.25	1.09–5.0	0.029
BUN (mg/dL)	6.8 (3.3; 2.1–20.0)	8.7 (6.0; 3.5–18.0)	1.25	1.12–1.39	<0.001
Total bilirubin (mg/dL)	0.4 (0.3; 0.1–2.9)	0.4 (0.2; 0.2–1.9)	0.71	0.16–3.06	0.643
Protein (g/L)	71.2 (6.2; 44.3–87.2)	71.5 (10.3; 57.1–80.4)	0.97	0.90–1.05	0.454
Albumin (g/L)	44.4 (4.8; 20.2–56.6)	42.6 (6.0; 34.2–48.0)	0.89	0.81–0.98	0.016
AP (U/L)	64.0 (26.0; 16.0–186.0)	71.5 (31.8; 44.0–141.0)	1.02	1.00–1.03	0.018
AST (U/L)	23.0 (11.0; 8.0–117.0)	25.0 (8.0; 12.0–263.0)	1.02	1.00–1.03	0.022
ALT (U/L)	27.0 (20.0; 7.0–230.0)	22.0 (9.0; 10.0–162.0)	0.99	0.98–1.02	0.923
GGT (U/L)	29.0 (29.0; 5.0–452.0)	41.5 (83.0; 13.0–284.0)	1.01	1.00–1.01	0.015
LDH (U/L)	177.0 (48.0; 23.0–401.0)	207.0 (78.0; 134.0–701.0)	1.02	1.01–1.02	<0.001
Glucose (mg/dL)	94.0 (24.0; 25.0–359.0)	104.0 (62.0; 53.0–300.0)	1.01	1.00–1.02	0.005
HbA1c (IFCC, mmol/mol)	37.0 (8.0; 25.0–89.0)	39.0 (14.5; 27.0–96.0)	1.06	1.03–1.09	<0.001
Triglyceride (mg/dL)	123.0 (100.0; 23.0–4077)	133.5 (91.0; 51.0–5090.0)	1.00	1.00–1.01	0.014
Total cholesterol (mg/dL)	193.0 (58.0; 65.0–597.0)	202.0 (98.0; 77.0–726.0)	1.01	1.00–1.01	0.030
CRP (mg/dL)	0.2 (0.4; 0.0–24.6)	0.4 (0.5; 0.0–0.9)	0.90	0.49–1.68	0.748
TSH (mIU/L)	1.6 (1.2; 0.0–15.3)	2.1 (2.0; 0.4–7.4)	1.14	0.88–1.46	0.324

* Values for laboratory parameters are presented as median (IQR; min–max). ALT: alanine aminotransferase; AP: alkaline phosphatase; aPTT: activated partial thromboplastin time; AST: aspartate aminotransferase; BUN: blood urea nitrogen; CRP: C-reactive protein; GGT: gamma-glutamyl transferase; INR: international normalized ratio; LDH: lactate dehydrogenase; MCH: mean corpuscular hemoglobin; MCHC: mean corpuscular hemoglobin concentration; MCV: mean corpuscular volume; TSH: thyroid-stimulating hormone. Laboratory analyses were exploratory and based on available preoperative values. Sample sizes for individual laboratory parameters are reported in Supplementary Table S1.

**Table 4:** Final LASSO-selected variables with Firth-corrected logistic regression model for wound healing disorder (n = 1060).

Variable	OR	95% CI	*p*-Value
Age	1.03	1.00–1.07	0.033
Operative duration	1.01	1.01–1.02	<0.001
Arterial hypertension	2.28	0.96–6.04	0.063

Candidate variables entered into LASSO regression were age, operative duration, arterial hypertension, LOA ratio, number of operated segments, and diabetes mellitus. Variables selected by LASSO were subsequently entered into Firth-corrected logistic regression. LOA: lumbosacral operated area.

## Data Availability

The data analyzed in this study are available from the corresponding author upon reasonable request. The data are not publicly available due to ethical and privacy restrictions related to patient-level clinical data.

## Supplementary Materials

The following supporting information can be downloaded at: https://www.mdpi.com/article/10.3390/jcm15145467/s1, Figure S1: Calibration plot of the final Firth-corrected logistic regression model; Table S1: Availability of preoperative laboratory parameter.

## Abbreviations

The following abbreviations are used in this manuscript:

AUC	Area under the curve
BMI	Body mass index
CRP	C-reactive protein
LASSO	Least absolute shrinkage and selection operator
LOA	Lumbosacral operated area
ROC	Receiver operating characteristic
TSH	Thyroid-stimulating hormone
WHDs	Wound healing disorders
