# Supplementary material for: Clinical and Surgical Risk Factors for Wound Healing Disorders Following Non-Instrumented Lumbar Spine Surgery Using Penalized Regression Analysis
Source: J Clin Med. 2026 Jul 13;15(14):5467. doi: 10.3390/jcm15145467 (PMC13412080; doi:10.3390/jcm15145467)
Supplement: Supplementary file 1 [file jcm-15-05467-s001.zip › Supplementary Table S1.pdf]

Supplementary Table S1. Availability of preoperative laboratory parameters.

Laboratory parameter	Available in No WHD	Available in WHD	Total available
Erythrocyte count (T/L)	938/1235	25/34	963/1269
Hemoglobin (g/dL)	938/1235	25/34	963/1269
Hematocrit (%)	938/1235	25/34	963/1269
MCV (fL)	938/1235	25/34	963/1269
MCH (pg)	938/1235	25/34	963/1269
MCHC (g/dL)	938/1235	25/34	963/1269
Platelet count (G/L)	938/1235	25/34	963/1269
Leucocyte count (G/L)	938/1235	25/34	963/1269
Prothrombin time (%)	850/1235	23/34	873/1269
INR	846/1235	23/34	869/1269
aPTT (sec)	901/1235	25/34	926/1269
Fibrinogen (mg/dL)	913/1235	25/34	938/1269
Sodium (mmol/L)	927/1235	23/34	950/1269
Potassium (mmol/L)	859/1235	20/34	879/1269
Creatinine (mg/dL)	934/1235	26/34	960/1269
BUN (mg/dL)	934/1235	26/34	960/1269
Total bilirubin (mg/dL)	747/1235	21/34	768/1269
Protein (g/L)	770/1235	22/34	792/1269
Albumin (g/L)	768/1235	22/34	790/1269
AP (U/L)	754/1235	22/34	776/1269
AST (U/L)	743/1235	21/34	764/1269
ALT (U/L)	770/1235	22/34	792/1269
GGT (U/L)	758/1235	22/34	780/1269
LDH (U/L)	712/1235	20/34	732/1269
Glucose (mg/dL)	907/1235	23/34	930/1269
HbA1c (IFCC, mmol/mol)	614/1235	16/34	630/1269
Triglyceride (mg/dL)	744/1235	22/34	766/1269
Total cholesterol (mg/dL)	665/1235	19/34	684/1269
CRP (mg/dL)	922/1235	24/34	946/1269
TSH (mIU/L)	658/1235	17/34	675/1269

ALT: alanine aminotransferase; *AP:* alkaline phosphatase; *aPTT:* activated partial thromboplastin time; *AST*: aspartate aminotransferase; *BUN*: blood urea nitrogen; *CRP*: C-reactive protein; *GGT*: gamma-glutamyl transferase; *INR*: international normalized ratio; *LDH*: lactate dehydrogenase; *MCH*: mean corpuscular hemoglobin; *MCHC*: mean corpuscular hemoglobin concentration; *MCV*: mean corpuscular volume; *TSH*: thyroid-stimulating hormone.
