# Supplementary figures and images for: Clinical and Surgical Risk Factors for Wound Healing Disorders Following Non-Instrumented Lumbar Spine Surgery Using Penalized Regression Analysis
Source: J Clin Med. 2026 Jul 13;15(14):5467. doi: 10.3390/jcm15145467 (PMC13412080; doi:10.3390/jcm15145467)

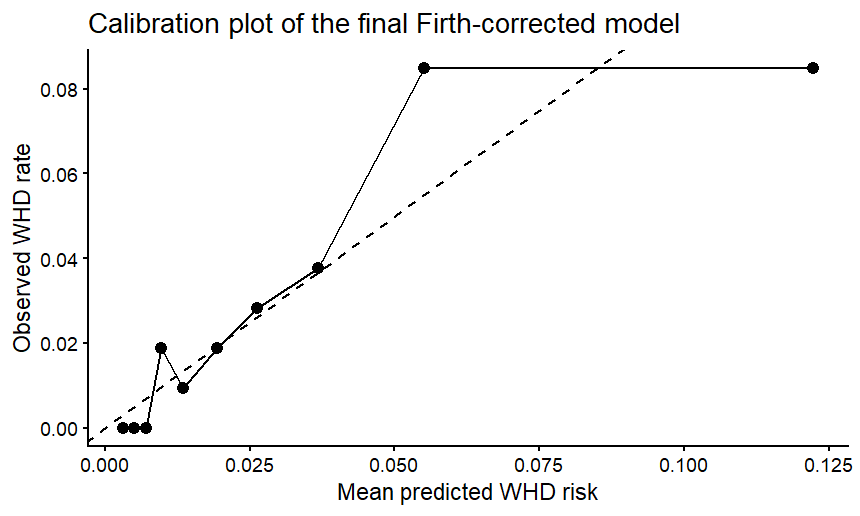

Supplement: Supplementary file 1 [file jcm-15-05467-s001.zip › Supplementary Figure S1. calibration plot.tiff]
